# The Impact of Mindfulness-Based Stress Reduction on Emotional Wellbeing and Glycemic Control of Patients with Type 2 Diabetes Mellitus

**DOI:** 10.1155/2018/1986820

**Published:** 2018-06-10

**Authors:** A. Armani Kian, B. Vahdani, A. A. Noorbala, A. Nejatisafa, M. Arbabi, S. Zenoozian, M. Nakhjavani

**Affiliations:** ^1^Department of Psychiatry, Beheshti Hospital and Zanjan University of Medical Sciences, Zanjan 45136-15788, Iran; ^2^Department of Psychiatry, Psychosomatic Medicine Research Center, Tehran University of Medical Sciences, Tehran, Iran; ^3^Department of Clinical Psychology, Beheshti Hospital and Zanjan University of Medical Sciences, Zanjan, Iran; ^4^Department of Endocrinology, Metabolism Research Center, Tehran University of Medical Sciences, Tehran, Iran

## Abstract

**Objective:**

The aim of the study was to determine the effect of mindfulness-based stress reduction (MBSR) intervention on emotion regulation and glycemic control of patients with type 2 diabetes.

**Materials and Methods:**

Sixty patients with type 2 diabetes were recruited for this randomized controlled trial from an outpatient clinic at Imam Hospital in Iran. The intervention group participated in 8 sessions of MBSR, and the control group continued the treatment as usual. Fasting blood sugar and HbA1c were measured as two indices of glycemic control. Overall mental health, depression, and anxiety were measured using the General Health Questionnaire (GHQ-28), Hamilton Depression Rating Scale (HDRS), and Hamilton Anxiety Rating Scale (HARS), respectively. All the assessments were performed at baseline and after 8 weeks and 3 months as follow-up.

**Results:**

In comparison with the control group, the MBSR intervention group showed a significant reduction on all outcome measures including FBS, HbA1C, HARS, and HDRS scores (*p* < 0/05).

**Conclusion:**

MBSR had a remarkable improvement on emotional wellbeing and glycemic control of patients with type 2 diabetes.

## 1. Introduction

Diabetes is a chronic metabolic multifaceted health problem, which is a common cause of death and disability in the whole world. It has significant effects not only on the body but also on the mind and social functioning. Affected patients with diabetes are 25.8 million people in the United States and approximately 8.3 percent of the population in 2011, according to the National Diabetes Clearinghouse.

Patients' quality of life, wellbeing, and social relationship get influenced by diabetes and its side effects [1–6]. According to the American Psychological Association (APA), 31% of patients with a chronic disorder tend to discuss about stress management methods with their health care provider, while only 19% put it in practice [7]. Nowadays, most people with diabetes suffer from stress mentally rather than physically, though the response of the body is the same to both kinds of stress, which leads to glycemic dysregulation [8]. The Mindfulness-Based Stress Reduction (MBSR) program is a technique, developed by Jon Kabat-Zinn in 1979 [9]. This approach is utilized to treat various chronic disorders such as anxiety, depression, pain, cancer, skin diseases, immune disorders, and diabetes [10]. The concept of the “mindfulness theory” provides insight into how thoughts and emotions impact our health, emotional wellbeing, and quality of life.

Over 200 medical centers all over the world offer MBSR as an alternative treatment option to the patients [11]. Hölzel et al. [12] stated that in an 8-week MBSR, a patient learns how to focus on a specific target, which causes changes in some specific regions of the patient brain associated to his/her memory, sense of self, empathy, and stress.

The goal of current study is to determine whether the MBSR therapy can help Iranian patients with diabetes to improve their physical and mental conditions.

## 2. Methods and Materials

### 2.1. Study Procedure

In a randomized clinical trial, 60 adult patients suffering from diabetes type 2 were recruited from outpatient endocrine clinics between April 2015 and June 2016. The hemoglobin A1c of all patients in the current study was higher than 7. Patients suffering from other chronic diseases, except hypertension, and patients with a history of treatment for psychiatric disorders or substance-related problems were excluded from this research. The patients were randomly assigned into the intervention and control groups. The intervention group participated in 8 sessions of MBSR, and the control group continued their treatment as 8 weekly usual visits. In the intervention group, participants with more than two session absences were also excluded. All participants completed written informed consent before entering the study, which was conducted under protocols approved by Imam Khomeini Hospital and Zanjan University of Medical Sciences ethics committees (Zums.rec.1396.41). The trial was registered in the Iranian Clinical Trials Registry, with registration number IRCT2017050925812N2.

All mindfulness sessions were supervised by a certified instructor, who has possessed at least a 3-year professional experience. The instruction of MBSR sessions was prepared based on presented instructions in Praissman et al. [11] and Rahmani et al. [13]. Additionally, each session includes selective and sustained concentration practices, introduction of new concepts, dyad/triad/whole group reflection/sharing about techniques and experiences in and out of class, reflection on poetry or readings, mindful listening practice, and opportunity for questions [14].

Fasting blood glucose and HbA1c were measured as two indices of glycemic control. Overall mental health, depression, and anxiety were measured using the General Health Questionnaire (GHQ-28), Hamilton Depression Rating Scale (HDRS), and Hamilton Anxiety Rating Scale (HARS), respectively. All the assessments were performed at baseline and after 8 weeks and 3 months as follow-up. A repeated-measures ANOVA with post hoc analysis was used to test between-group differences.

### 2.2. Measures

#### 2.2.1. Demographic Information Questionnaire

This questionnaire is used to collect basic information including age, marital status, socioeconomic condition, educational background, patients' adherence, existing diabetes complications and comorbid conditions, alcohol consumption, and smoking.

#### 2.2.2. General Health Questionnaire-28

The GHQ-28 is a 28-item questionnaire used to measure emotional distress in medical settings in four subscales such as somatic symptoms (items 1–7), anxiety/insomnia (items 8–14), social dysfunction (items 15–21), and severe depression (items 22–28). According to a previous study, the reliability coefficient of the test ranges from 0.78 to 0.95.

#### 2.2.3. Hamilton Depression Rating Scale

It is designed to assess transient and fluctuating mood status based on the clinician's interview with a patient to evaluate symptoms such as depressed mood, hopelessness, suicide ideation, sleep disturbances, anxiety levels, and weight loss.

#### 2.2.4. Hamilton Anxiety Rating Scale

This widely used interview scale measures the extremity of a patient's anxiety, based on 14 parameters including anxious mood, tension, fears, insomnia, somatic complaints, and behavior in the client's interview. Test-retest reliability and internal consistency for Hamilton anxiety were assessed as 0.86 (CI 95% 0.78–0.91) and 0.85, respectively [15].

## 3. Results

Sixty patients with type 2 diabetes participated in the study. Thirty patients (27 females and 3 male) were in the control group, and 30 patients (22 females and 8 male) were in the MBSR intervention group. A female patient in the intervention group resigned after the first session. The demographic and baseline characteristics of the participant are presented in Table 1, and differences between the control and MBSR group were not significant.

**Figure 1:**
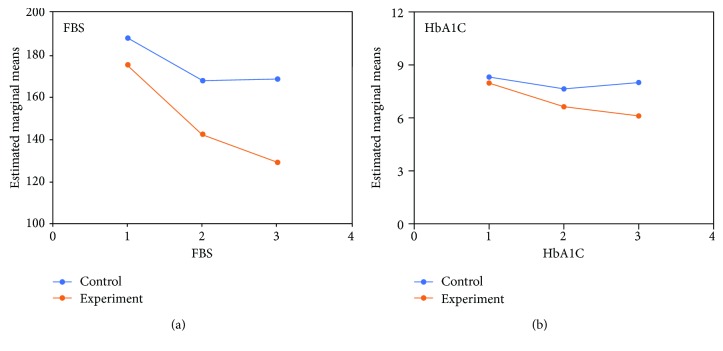
The trajectory of changes in glycemic control measures (FBS and HbA1c) in the intervention and control groups during the time of study (1 = baseline, 2 = 8 weeks, 3 = 3 months).

**Figure 2:**
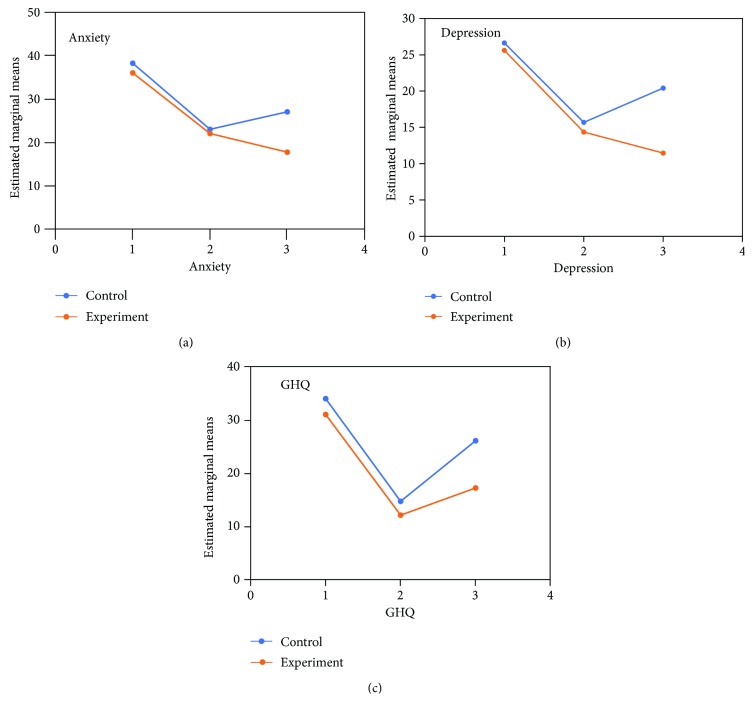
The trajectory of changes in mental health measures (GHQ-28 score, HARS score, and HDRS score) in the intervention and control groups during the time of study (1 = baseline, 2 = 8 weeks, 3 = 3 months).

The trend for changes in glycemic control measures (FBS and HbA1c) and mental health measures (GHQ-28 score, HARS score, and HDRS score) is illustrated in Figures 1 and 2, respectively.

### 3.1. Glycemic Control Measures

#### 3.1.1. Fasting Blood Sugar

The difference between the two groups was significant as indicated by the effect of group, the between-subject factor (Huynh-Feldt correction; df = 2, *F* = 99.12, *P* = 0.0001). The behavior of the two arms of treatment was not similar across time (group-by-time interaction, Huynh-Feldt correction; *F* = 8.64, df = 2, *P* = 0.001). In addition, a one-way repeated-measures analysis of variance showed a significant effect of both treatments on FBS (*P* < 0.001). In both groups, post hoc comparisons showed a significant change from baseline on the FBS. The difference between the two treatments was significant at the first endpoints (week 8; *t* = 3.28, df = 29, *P* < 0.001) and the second endpoint (3 months; *t* = 2.78, df = 29, *P* < 0.001).

#### 3.1.2. HbA1C

The difference between the two groups was significant as indicated by the effect of group, the between-subject factor (Huynh-Feldt correction; df = 2, *F* = 69.75, *P* = 0.0001). The behavior of the two arms of treatment was not similar across time (group-by-time interaction, Huynh-Feldt correction; *F* = 18.78, df = 2, *P* = 0.001). In addition, a one-way repeated-measures analysis of variance showed a significant effect of both treatments on HbA1C (*P* < 0.001). In both groups, post hoc comparisons showed a significant change from baseline on HbA1c. The difference between the two treatments was significant at the first endpoints (week 8; *t* = 2.94, df = 29, *P* < 0.01) and the second endpoint (3 months; *t* = 3.22, df = 29, *P* < 0.01).

### 3.2. Mental Health Measures

#### 3.2.1. GHQ-28 Score

The difference between the two groups was significant as indicated by the effect of group, the between-subject factor (Huynh-Feldt correction; df = 2, *F* = 60.70, *P* = 0.0001). The behavior of the two arms of treatment was not similar across time (group-by-time interaction, Huynh-Feldt correction; *F* = 3.67, df = 2, *P* = 0.02). In addition, a one-way repeated-measures analysis of variance showed a significant effect of both treatments on GHQ-28 score (*P* < 0.001). In both groups, post hoc comparisons showed a significant change from baseline on the GHQ-28 score. The difference between the two treatments arm was significant at the first endpoint (week 8; *t* = 3.42, df = 29, *P* < 0.001) and the second endpoint (3 months; *t* = 2.56, df = 29, *P* < 0.001).

#### 3.2.2. HDRS Score

The difference between the two groups was significant as indicated by the effect of group, the between-subject factor (Huynh-Feldt correction; df = 2, *F* = 53.71, *P* = 0.0001). The behavior of the two arms of treatment was not similar across time (group-by-time interaction, Huynh-Feldt correction; *F* = 3.81, df = 2, *P* = 0.001). In addition, a one-way repeated-measures analysis of variance showed a significant effect of both treatments on HDRS score (*P* < 0.001). In both groups, post hoc comparisons showed a significant change from baseline on HbA1c. The difference between the two treatments was significant only at the first endpoints (week 8; *t* = 3.68, df = 29, *P* < 0.01).

#### 3.2.3. HARS Score

The difference between the two groups was significant as indicated by the effect of group, the between-subject factor (Huynh-Feldt correction; df = 2, *F* = 105.33, *P* = 0.0001). The behavior of the two arms of treatment was not similar across time (group-by-time interaction, Huynh-Feldt correction; *F* = 4.78, df = 2, *P* = 0.01). In addition, a one-way repeated-measures analysis of variance showed a significant effect of both treatments on HARS score (*P* < 0.001). In both groups, post hoc comparisons showed a significant change from baseline on the HARS score. The difference between the two treatment arms was significant, just at the first endpoint (week 8; *t* = 2.35, df = 29, *P* < 0.01).

## 4. Discussion

The finding of the current study embarked that the use of MBSR programs increases the wellbeing and general health, while it decreases FBS, HbA1c, anxiety, and depression of patients with type 2 diabetes.

A systematic review about the effect of MBSR on patients with diabetes was conducted in 2014, but failed to show the presence of any benefit related to glycemic control [8]. Another review performed by Noordali et al. [16] attempted to assess the benefits of mindfulness-based interventions in patients with either type 1 or type 2 diabetes. A total of 11 studies met the researchers' inclusion criteria. In this report, four studies found that mindfulness-based stress interventions alleviated patients' HbA1c levels, though another three surveys (which employed larger sample sizes, maintained higher levels of quality, and enjoyed less risk of bias) found no such change.

A research conducted by Rosenzweig et al. found a decrease in HbA1c levels. The findings also showed improvements in participants' stress management practices, emotional distress levels, and quality of life [17].

Furthermore, studies conducted by Hartmann et al. [18] and Teixeira [19] showed that mindfulness meditation and stress reduction could improve the psychological coping skills of diabetic patients.

Mindfulness techniques elicited improvements to patients' stress, anxiety, and depression symptoms in patients with diabetes; such improvements were similar to those found when MBSR was applied to other chronic illnesses [10, 20, 21]. Moreover, Keyworth et al. [22] noted improvements in levels of worry and thought suppression.

Various factors can influence mindfulness interventions that may play a confounding role and should be considered as limitations of the present study. Mindful eating techniques, introduced in an effort to develop an individual understanding of diet and nutrition and to increase patients' mindful awareness with regard to eating, were effective in achieving favorable results [23, 24]. Self-report questionnaires and measurements are other factors that require additional attention if researchers hope to prevent bias. As such, further study is needed to obtain additional information regarding specific effects of MBSR on patients with diabetes.

## 5. Conclusions

MBSR is a useful method to help patients with type 2 diabetes to diminish their emotional distress and to improve glycemic control.

## Figures and Tables

**Table 1:** Baseline demographic and clinical characteristics of the sample of patients with type 2 diabetes who were participated in the RCT.

Characteristics	MBSR	Control	*P* value
Age, mean	53.48	59.03	0.13
Gender, *n* (%)			
Male	8 (27.5)	3 (10)	0.23
Female	21 (72.4)	27 (90)	
Education level, *n* (%)			
Elementary school	5 (17.2)	1 (3.3)	0.14
High school	14 (48.2)	17 (56.6)	
Higher education	10 (34.4)	12 (40)	
Marital status, *n* (%)			
Single	4 (13.7)	3 (10)	0.085
Married	18 (62)	21 (70)	
Divorced	7 (24.1)	6 (20)	
Working, *n* (%)	12 (41.3)	11 (36.6)	0.91
Illness duration, mean (SD)	12 (6.2)	10 (4.5)	0.45
Complications, *n* (%)	16 (55.1)	13 (46.6)	0.39
Comorbidity, *n* (%)	17 (58.6)	13 (43.3)	0.27
Medication type, *n* (%)			
Metformin	9 (31)	15 (50)	0.09
Sulfonylureas	5 (17)	3 (10)	
Antidiabetic combination	15 (51)	12 (40)	
FBS	175.3 (44,5)	187.6 (59.6)	0.91
HbA1C	7.9 (1.1)	8.3 (1.7)	0.87
HARS	36 (11.4)	38.2 (9.6)	0.81
HDRS	26.5 (13.3)	27.5 (12.7)	0.29
GHQ	31 (9.5)	30.9 (13.2)	0.1

MBSR: mindfulness-based stress reduction group; FBS: fasting blood sugar; HbA1c: hemoglobin A1c. HARS: Hamilton Anxiety Rating Scale; HDRS: Hamilton Depression Rating Scale. GHQ: General Health Questionnaire.
